# Supplementary material for: Non-traditional fluorescence in quadruple hydrogen bonded supramolecular polymers
Source: Nat Commun. 2026 Jan 20;17:776. doi: 10.1038/s41467-025-67128-2 (PMC12823654; doi:10.1038/s41467-025-67128-2)
Supplement: Supplementary file 1 — Supplementary Information [file 41467_2025_67128_MOESM1_ESM.pdf]

Supplementary Information

Non-traditional Fluorescence in Quadruple Hydrogen Bonded Supramolecular Polymers

Han Zuo, Yi Zeng, Qinghua Gao, Zexiang Wang, Qiannan Zhang, Youliang Zhu, Xiaoyan Zheng, Chuancheng Jia*, Pingchuan Sun, Ben Zhong Tang*, Fenfen Wang **

Suppl. Note 1. Experimental Section

Detailed information of the raw materials: Polytetramethylene glycol (PTMG, Mn=2000 g/mol) Triethylamine (Et_3N), 2-acetylbutyrolactone, guanidine carbonate, were purchased from Sigma-Aldrich (Shanghai China). 1,6-hexyldiisocyanate (HDI, 99%) and dibutyltindilaurate (DBTDL) were obtained from J&K Scientific Ltd.PTMG-2000 sample was dried in vacuum at 120 °C for two hours prior to use. *N*,*N*-Dimethylformamide (DMF) was dried over CaH_2 prior to distillation under reduced pressure. All the other reagents were received from Tianjin Concord Pharmaceutical Chemical Co. Ltd, and used as received without further purification.

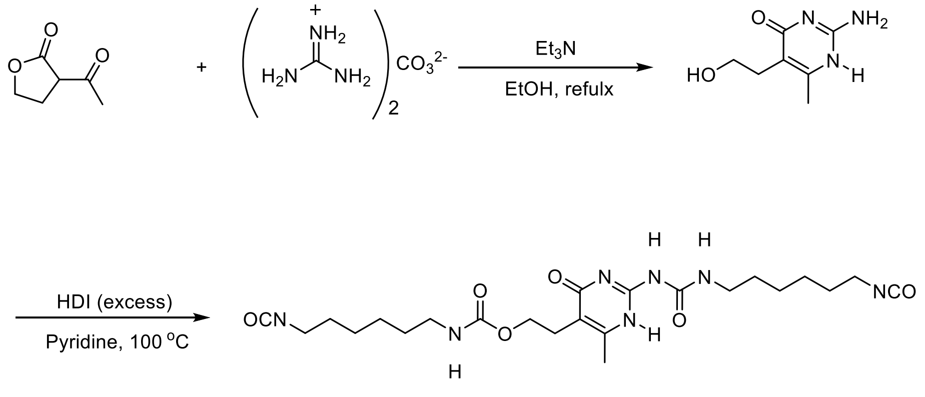

Supplementary Figure 1. Schematic synthesis pathway of UPy-DHDI.

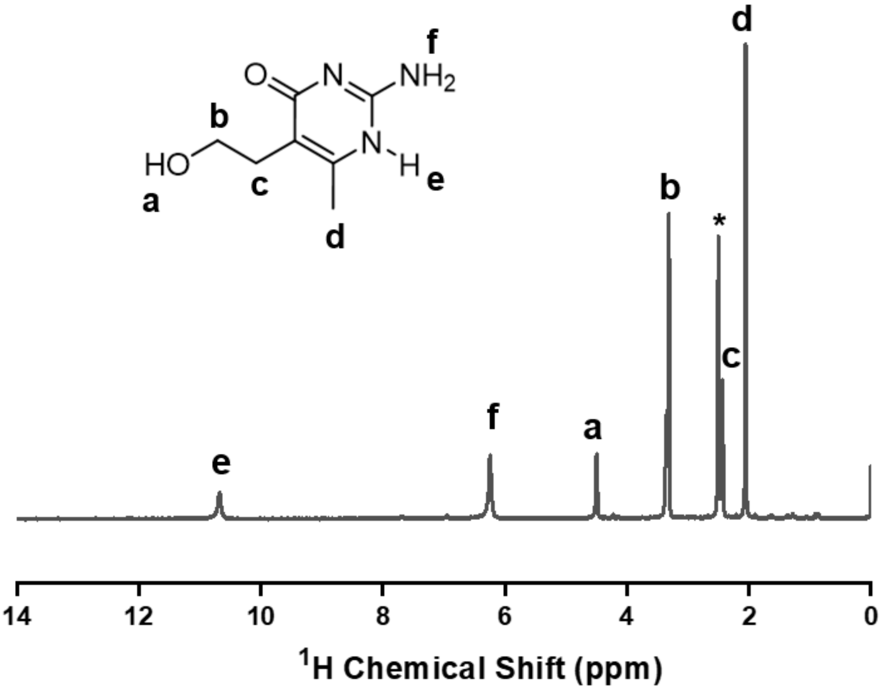

Supplementary Figure 2. ^{1}H NMR spectrum of UPy precusor in DMSO-d6. “*” denotes the proton signals of DMSO-d6.

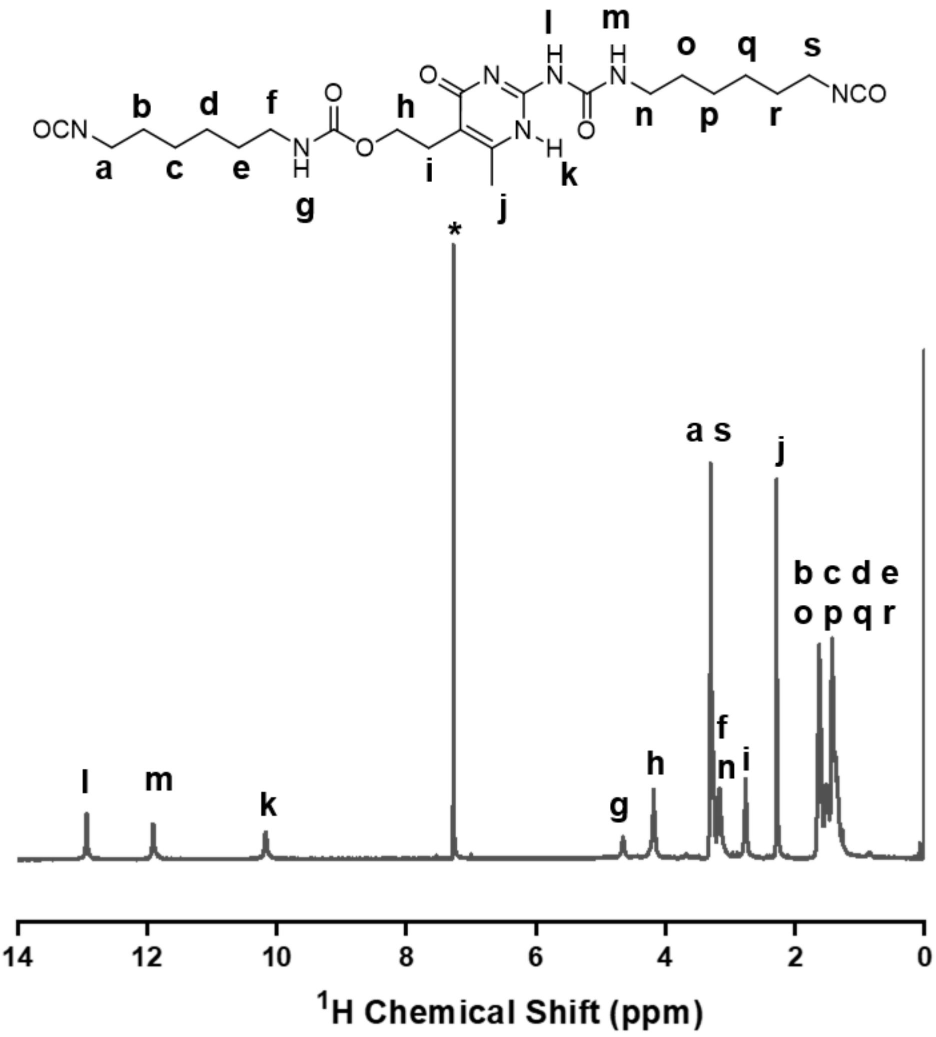

Supplementary Figure 3. ^{1}H NMR spectrum of UPy-DHDI in $CDCl_3$, “*” denotes the solvent signal.

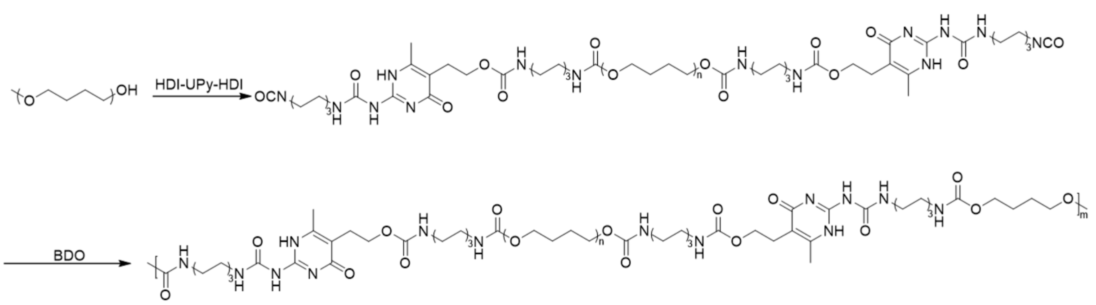

Supplementary Figure 4. Schematic synthesis pathway of PU-UPy.

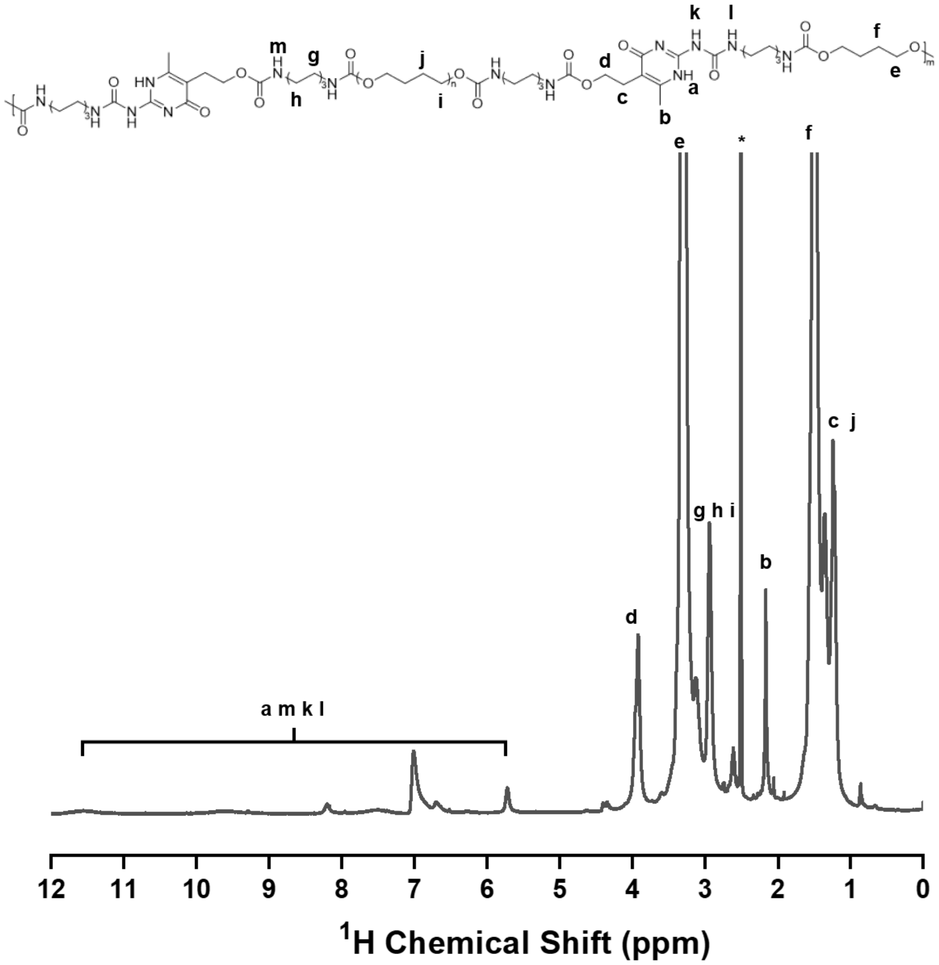

Supplementary Figure 5. ^{1}H NMR spectrum of UPy-DHDI inDMSO, “*” denotes the solvent signal.

Suppl. Note 2. Results and Discussion

Details of the cell experiments: The extract of PU-UPy was used to culture cells to access the biocompatibility of the material. Take a certain amount of DMEM medium and mix with 10% fetal bovine serum and 1% bispecific antibody (penicillin/streptomycin). Mouse fibroblast cell line L929 cells were selected and thawed in DMEM complete medium at 37°C, 5% CO_2 culture in a CO_2 incubator, then trypsinized after 3 passages, counted using a cell slide, and adjusted to 5000-8000 cells/mL. The experimental materials were cut at a 0.1g/mL extraction ratio

(mass/volume) ± 10% and sterilized by ultraviolet light. Then, the specimens were placed in a 50mL sterile centrifuge tube and 40mL of serum-free DMEM medium were added for extraction, and then extracted in a thermostatic shaker at 37±1 °C for 24 hours before setting aside. The blank values of the individual test kits are used as the blank group. Dilute the extract into 1, 2, 4, 6, 8, 10, 30, 50-fold with DMEM complete medium.6 parallel experiments were performed for each experimental group of cell cultures. 0.5mL of cell solution at a concentration of 5000-8000 cells/mL was added to each well of a 24-well culture plate and incubated in a CO_2 incubator at 37°C, 5% CO_2 for 24 hours. Pipette the medium out of the well plate, add 1mL of the culture medium of the experimental group, the control group, and the blank control group, and then put it into the CO_2 incubator and incubate it at a temperature of 37°C and a CO_2 concentration of 5% for 1, 3, and 5 days. DMEM medium was measured, and 10% CCK-8 reagent was added to obtain CCK-8 cell viability test solution. First, aspirate the original culture medium from the plate, wash it with PBS 3 times, and add 0.2mL of CCK-8 test solution to each well; Then, each experimental group was placed in a CO_2 incubator and incubated at 37°C, 5% CO_2 for 2h. Finally, 0.1 mL of the incubated test solution was aspirated and added to a 96-well plate, and its absorbance (OD value) at 450 nm was determined by a microplate reader, and the absorbance value was converted into a relative growth rate.

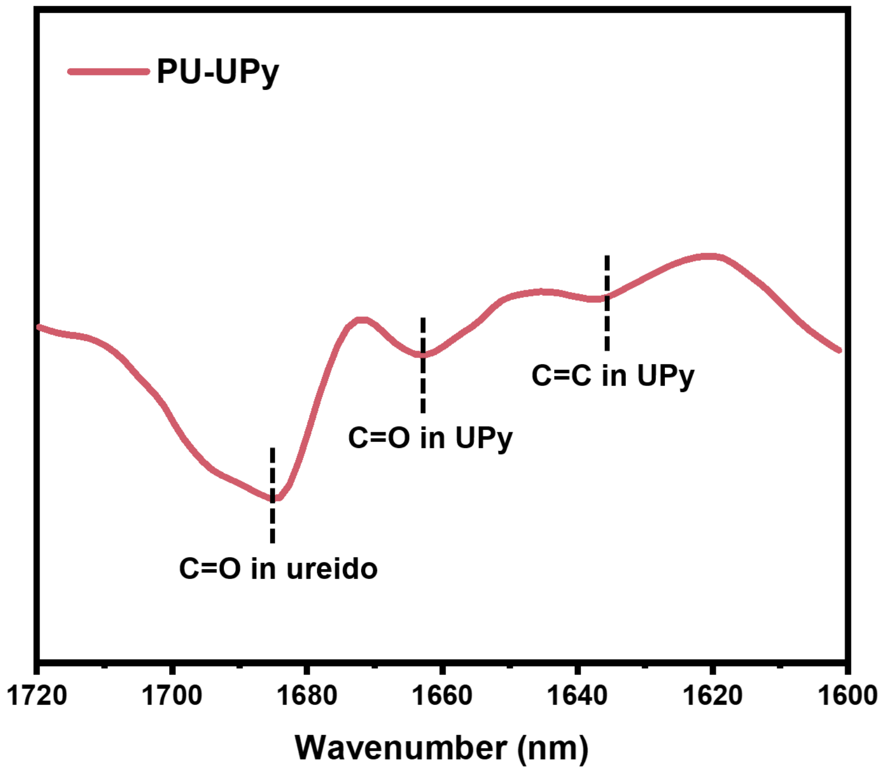

Supplementary Figure 6. FTIR spectra of PU-UPy.

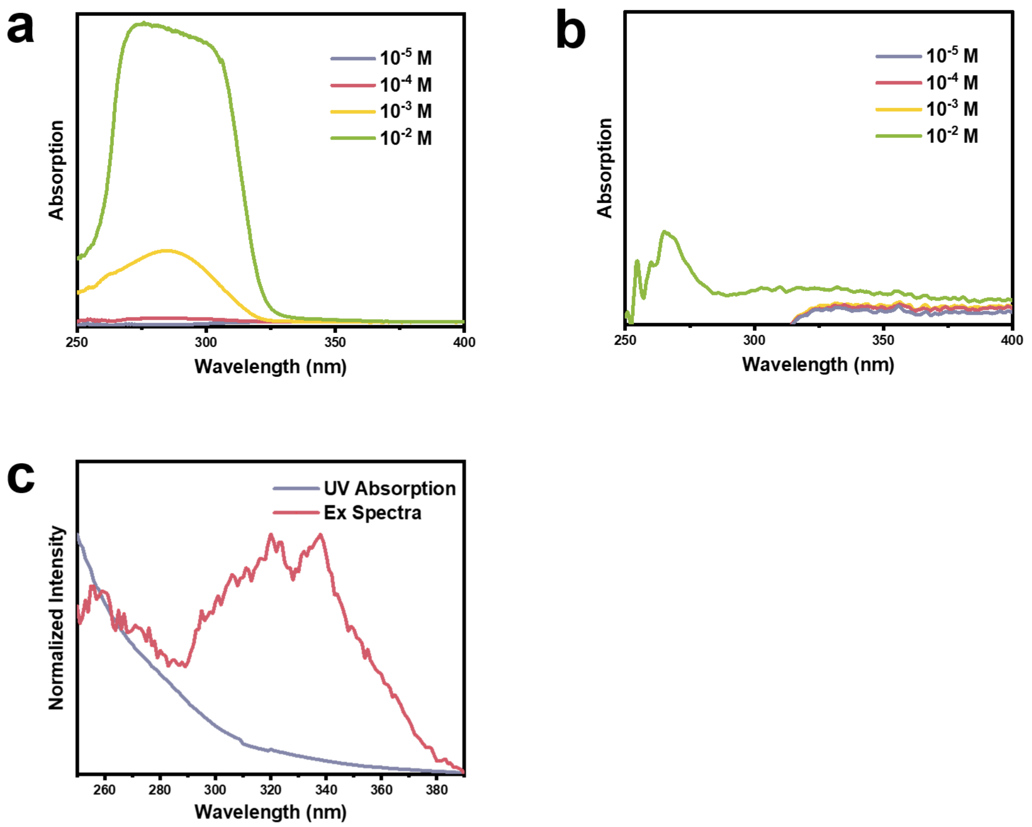

Supplementary Figure 7. UV−vis spectra of PU-UPy (a) and PU-b (b) in DMF. (c) Normalized UV−vis spectra and excitation spectra of PU-b (solid state).

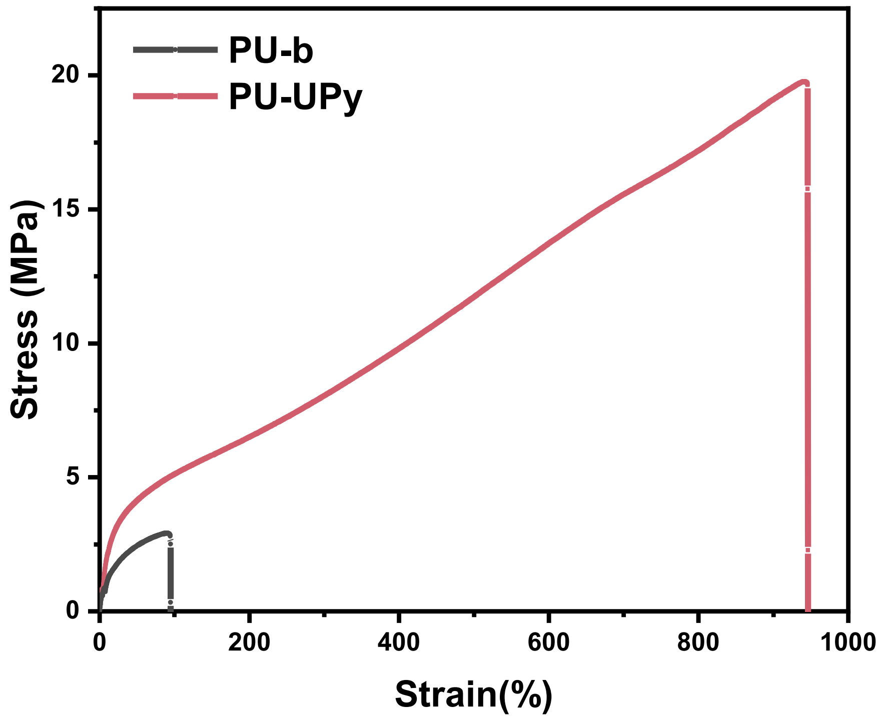

Supplementary Figure 8. Stress−strain curves of PU-UPy obtained from tensile test at a stretching rate of 50 mm/min.

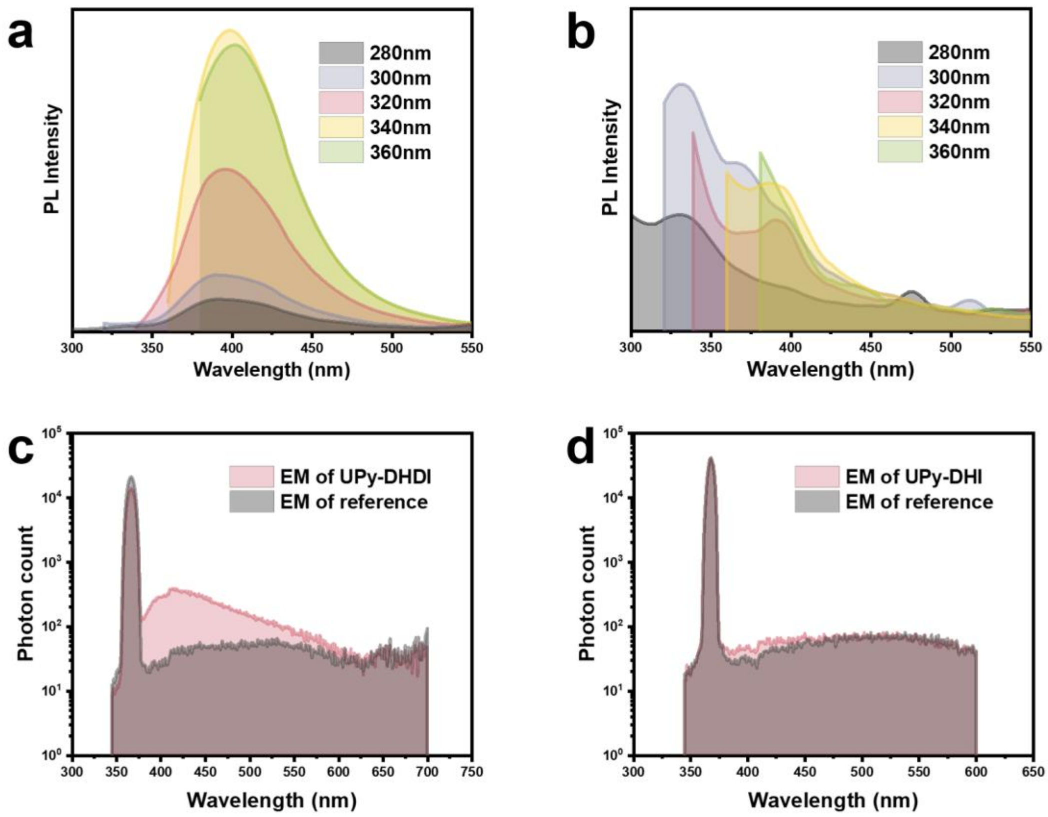

Supplementary Figure 9. Emission spectra of UPy-DHDI (a) and UPy-DHI (b) at solid state, excited at different wavelength. PLQY test results of UPy-DHDI (c) and UPy-DHI (d).

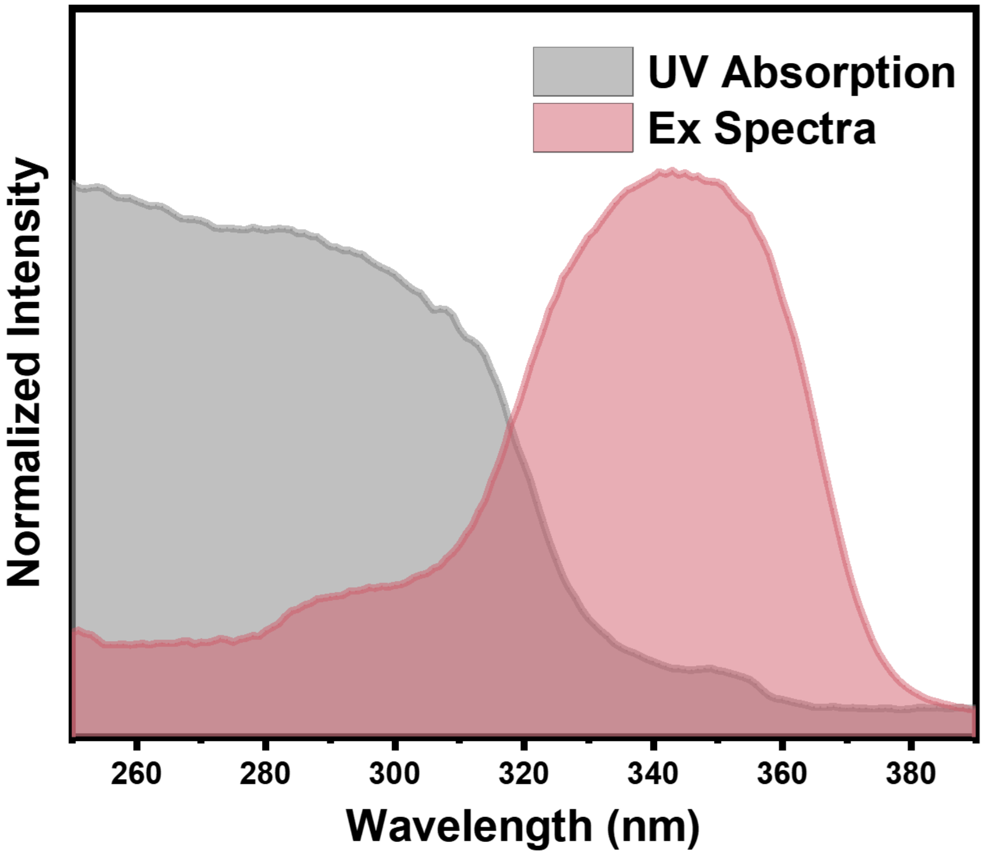

Supplementary Figure 10. Normalized UV absorption and excitation spectra of solid UPy-DHDI.

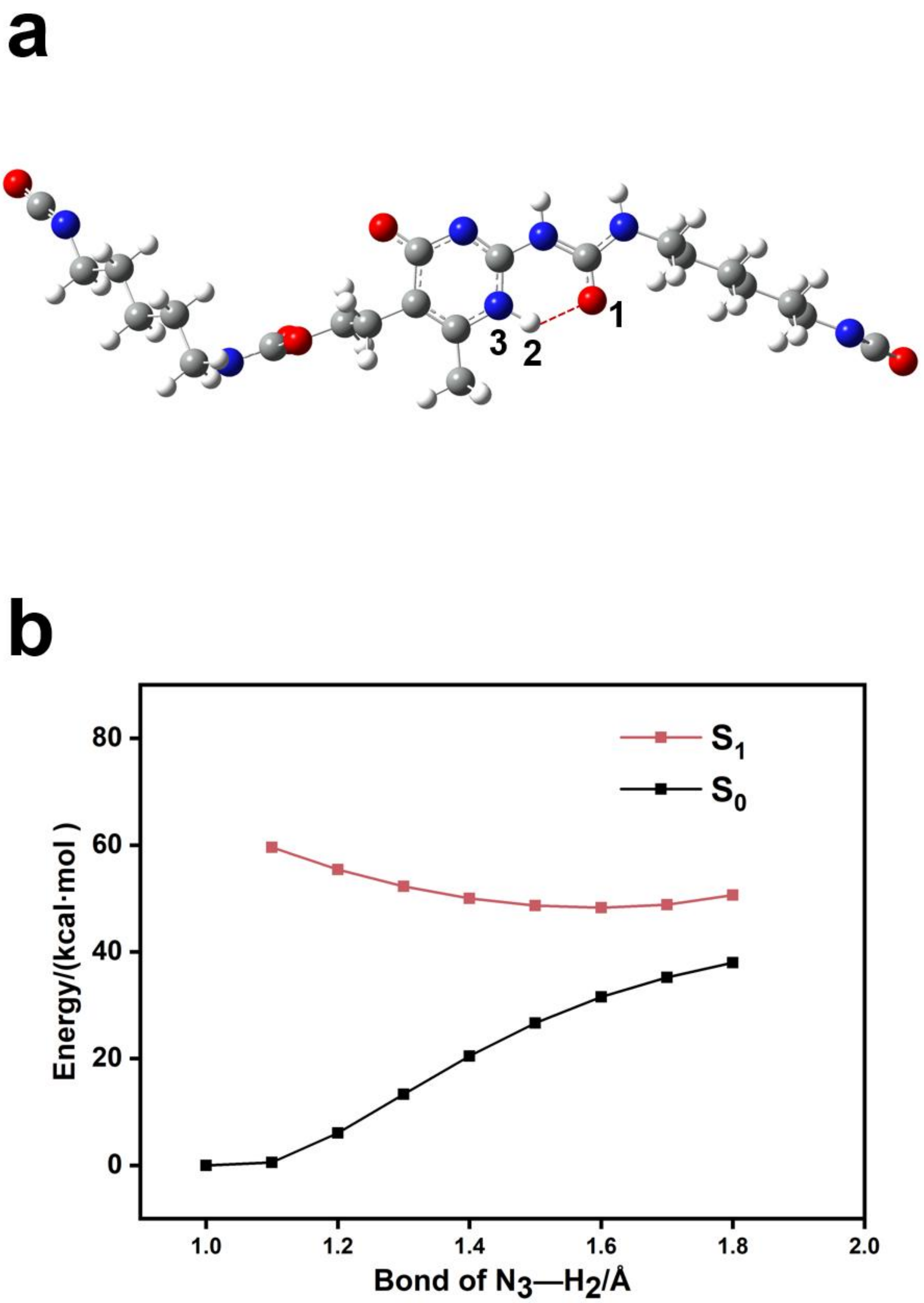

Supplementary Figure 11. (a) Optimized structure of UPy-DHDI (Labels1,2,3 mark the N,O, and C atoms participating in ESPT). (b) The potential energy curve of ESPT product.

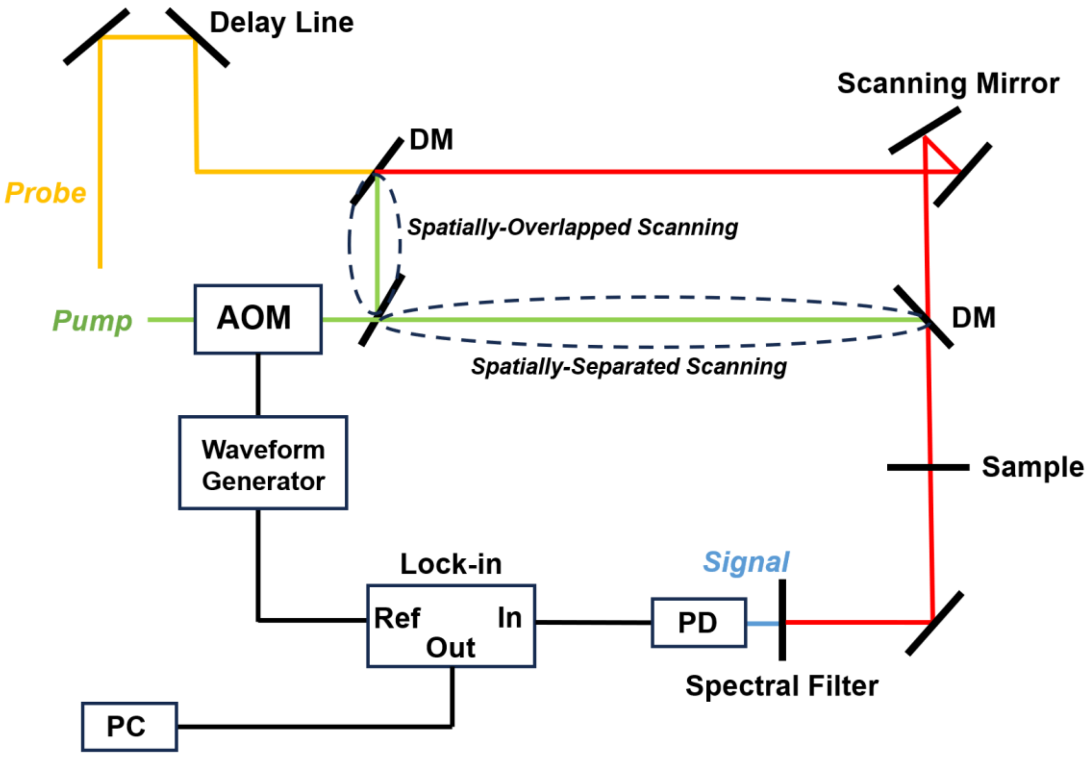

Supplementary Figure 12. Illustration of the transient absorption optical path.

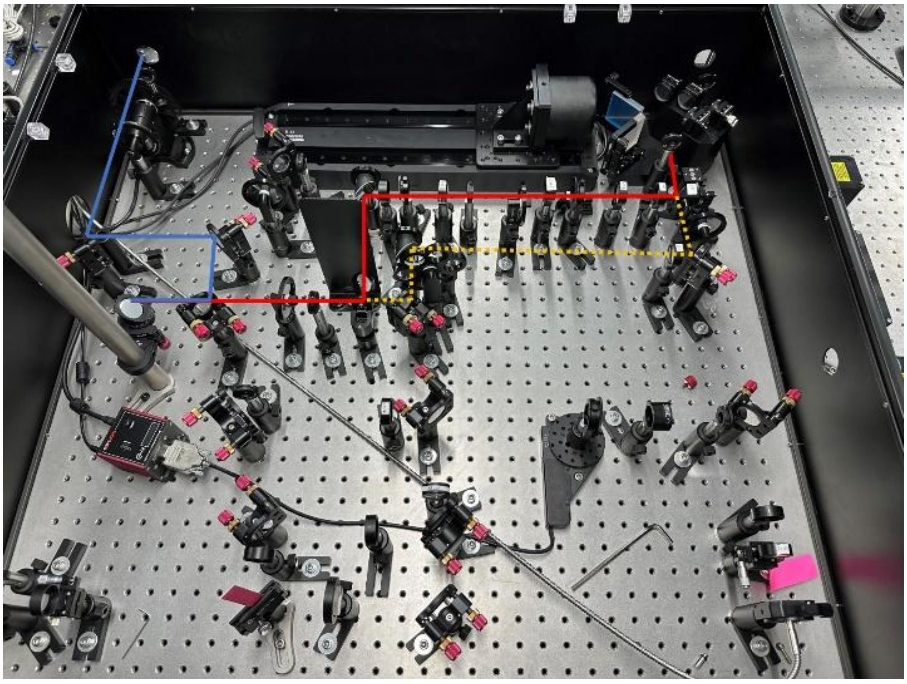

Supplementary Figure 13. Photograph of transient absorption optical path.

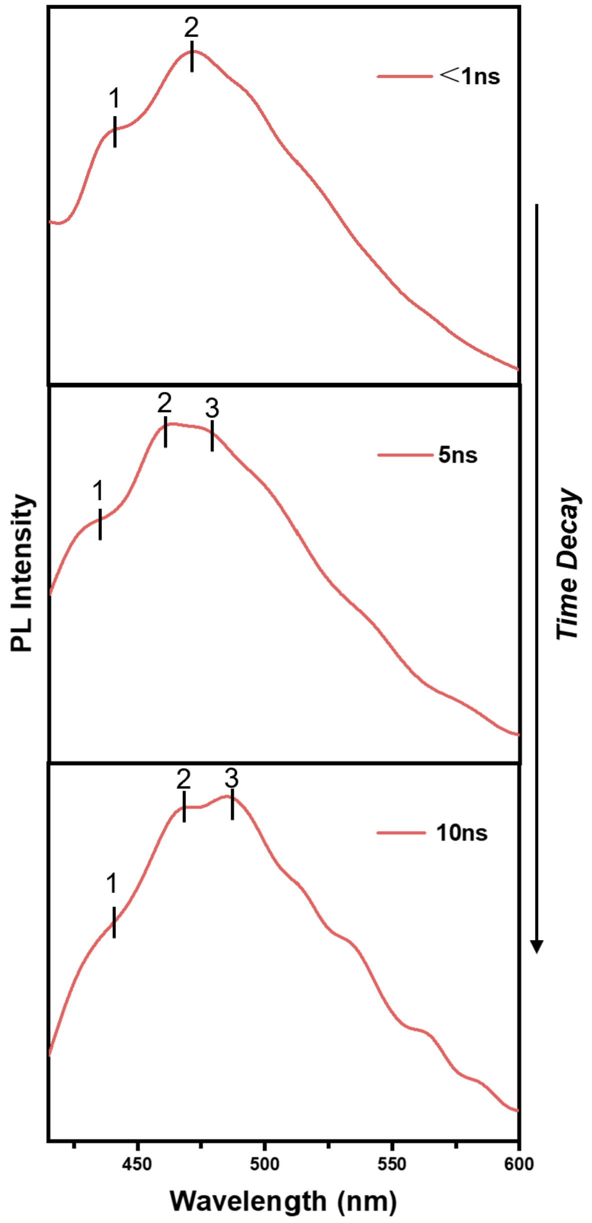

Supplementary Figure 14. Time-resolved emission spectrum of PU-UPy. Peaks 1, 2, and 3 correspond to three distinct emission peaks, which are attributed to the Keto configuration, Enol1 configuration, and Enol2 configuration, respectively).

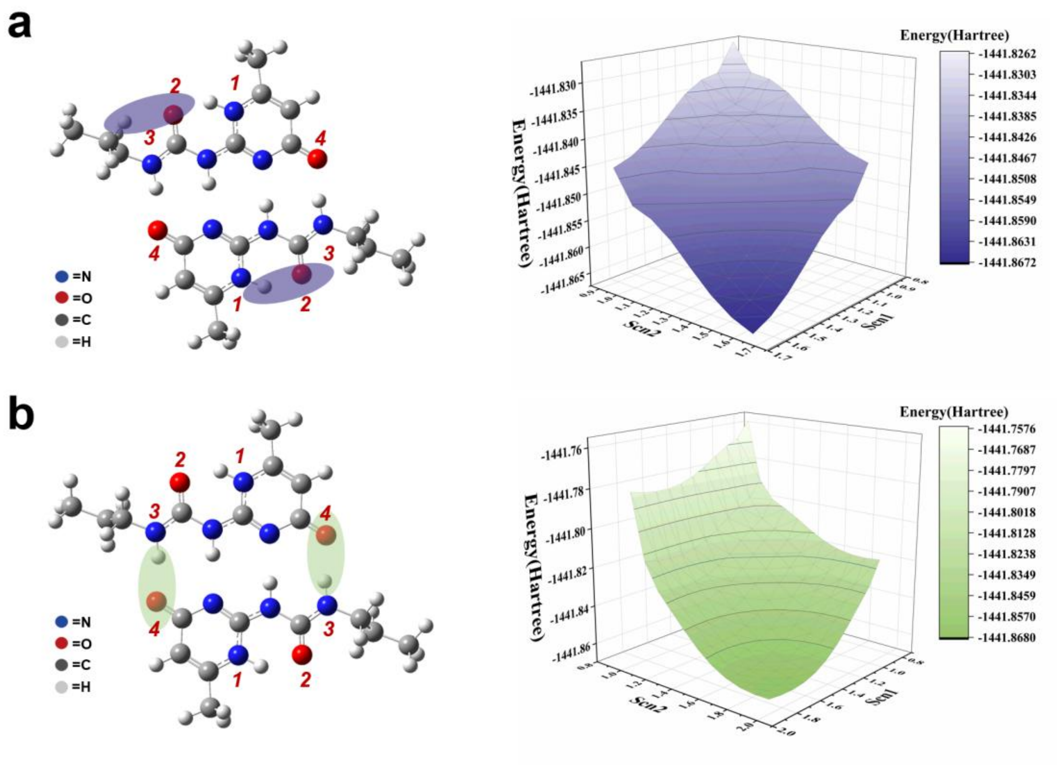

Supplementary Figure 15. Potential energy surface scanning for intramolecular (a) intermolecular (b) ESPT (The color-coded blocks in the structural formula designate the sites of proton transfer).

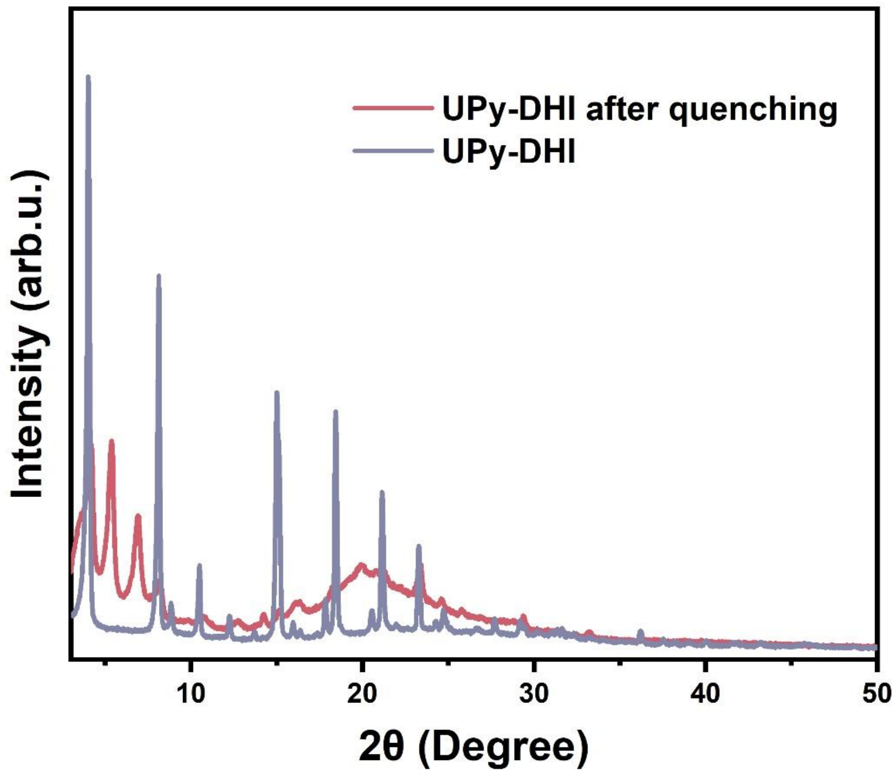

Supplementary Figure 16. XRD patterns of UPy-DHI before and after quenching treatment.

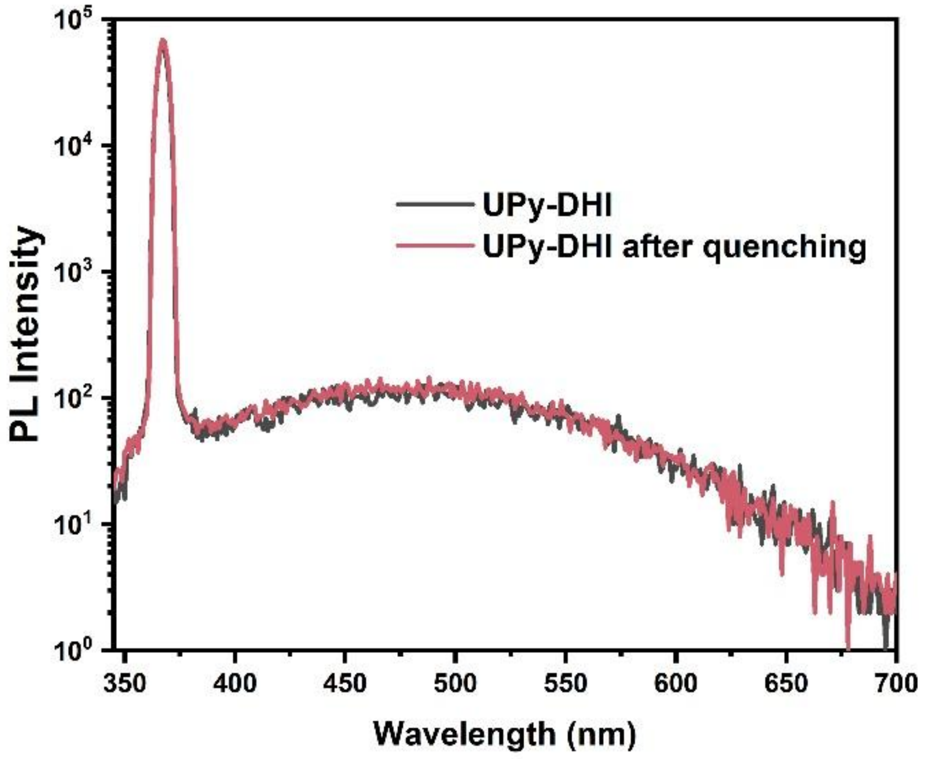

Supplementary Figure 17. PLQY testing of UPy-DHI before and after quenching treatment.

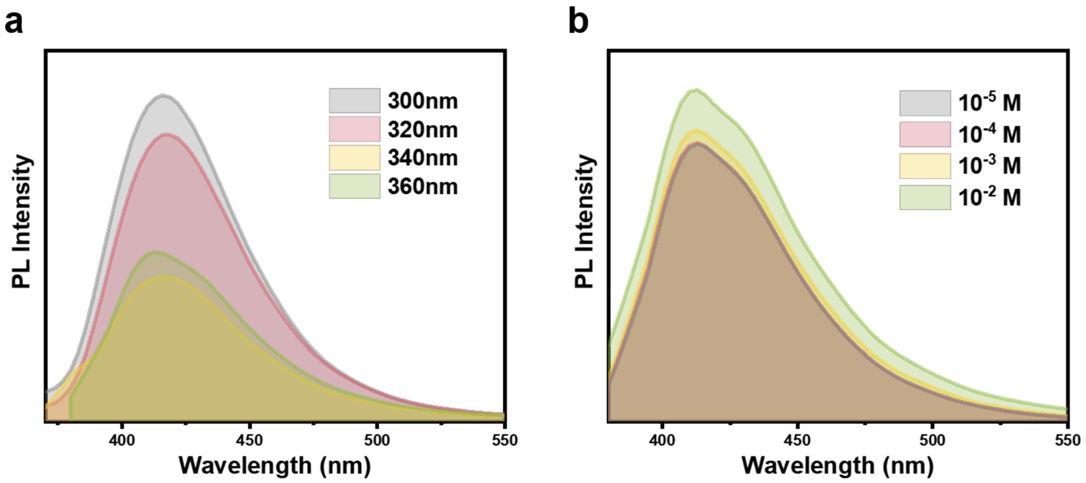

Supplementary Figure 18. (a) Emission spectra of UPy-DHI chloroform solution at different excitation wavelengths (0.01M). (b) Emission spectra of UPy-DHI chloroform solutions at different concentrations (excited at 360 nm).

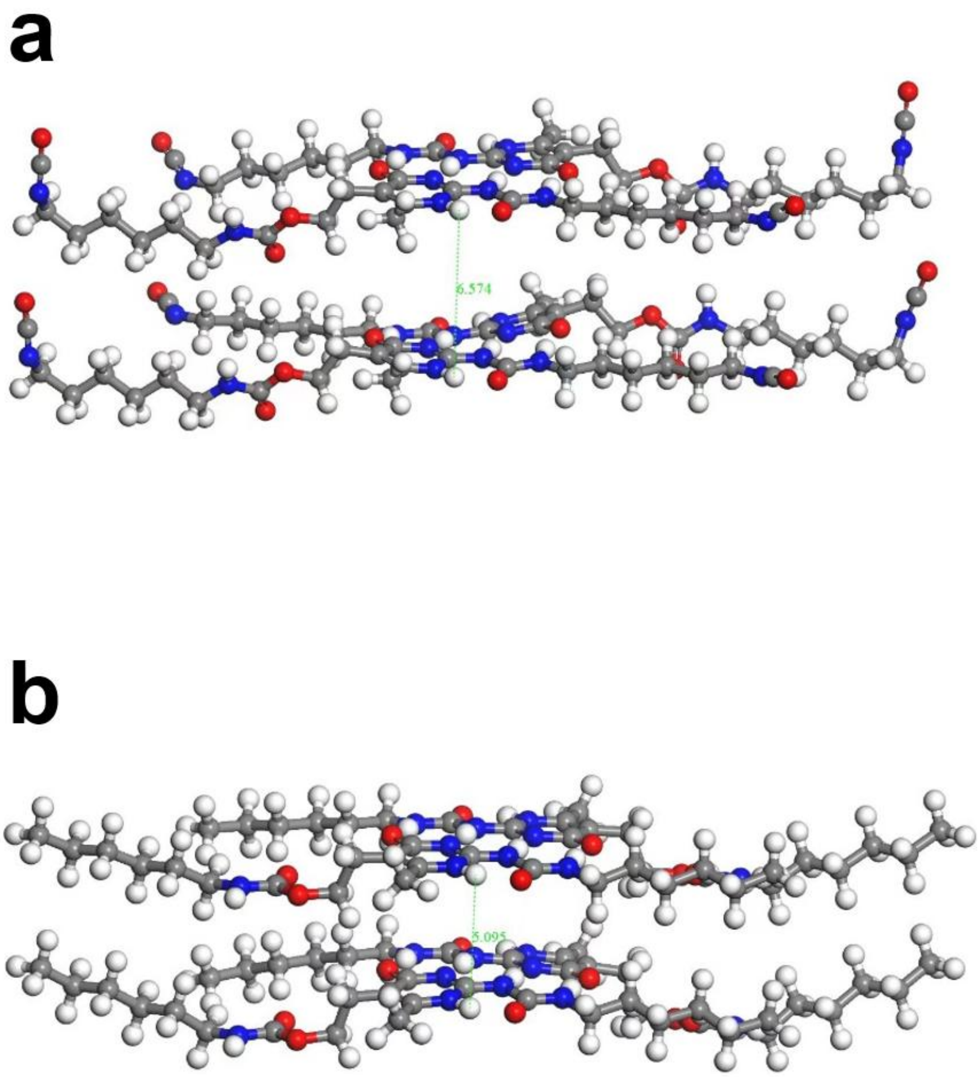

Supplementary Figure 19. UPy structure simulated by Gaussian of UPy-DHDI (a) and UPy-DHI (b).

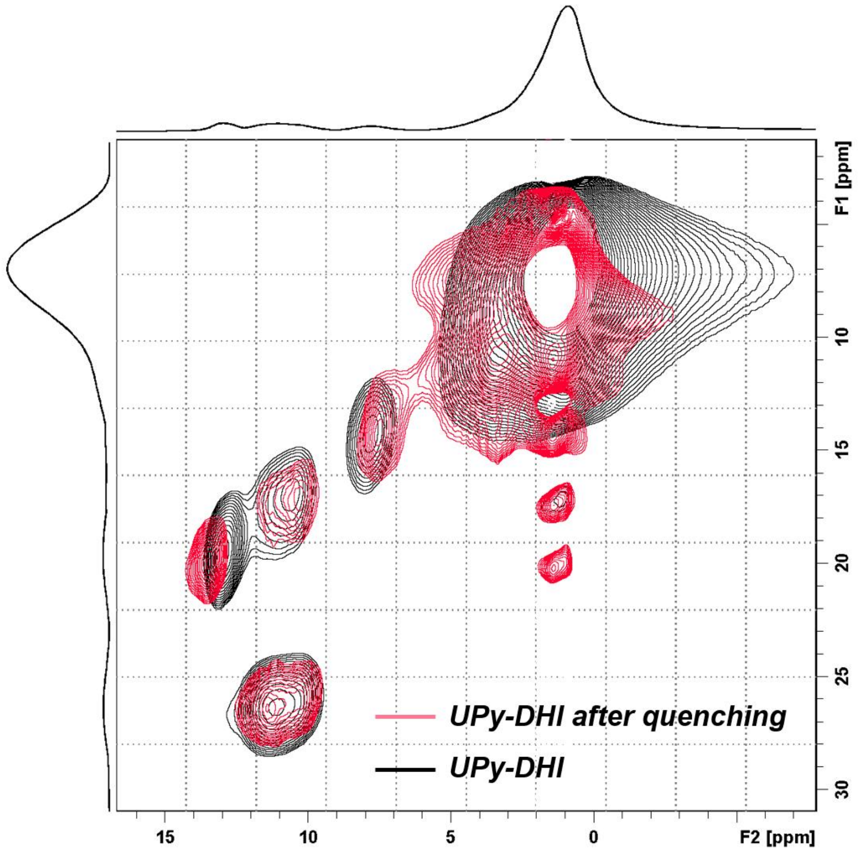

Supplementary Figure 20. Solid-state NMR two-dimensional DQ/SQ correlation spectra of UPy-DHI sample before and after quenched in liquid nitrogen.

Supplementary Table 1. T_1 relaxation time of ^{13}C nucleus in UPy core.

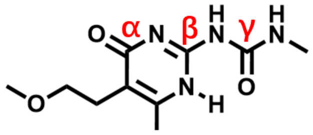

items	Sample	α	β	γ
T_1C (s)	UPy-DHI	100	106	129
	UPy-DHDI	46.7	54.7	36.8

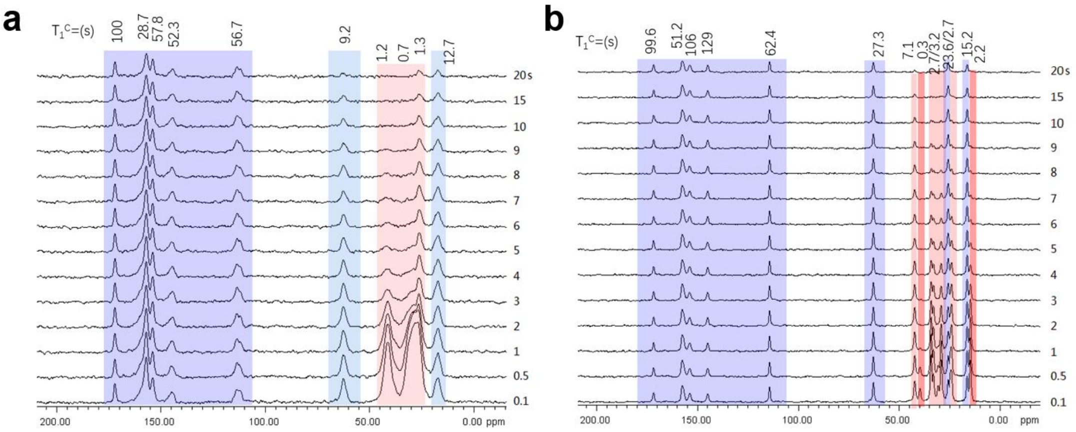

Supplementary Figure 21. Solid-state NMR relaxation time (T_1) spectrum of UPy-DHDI (a) and UPy-DHI (b).

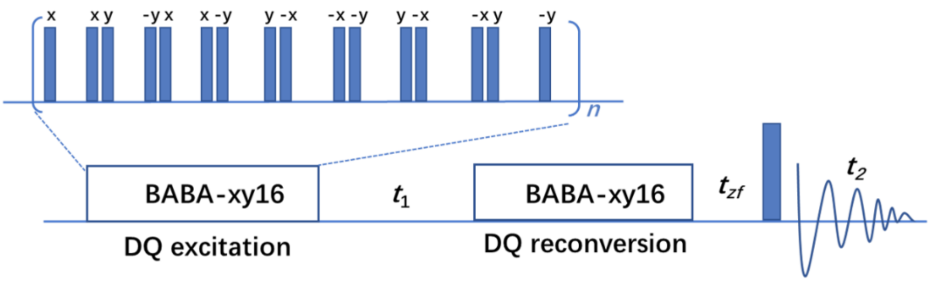

Supplementary Figure 22. Pulse sequences for obtaining 2D proton DQ/SQ spectrum under ultrafast MAS.

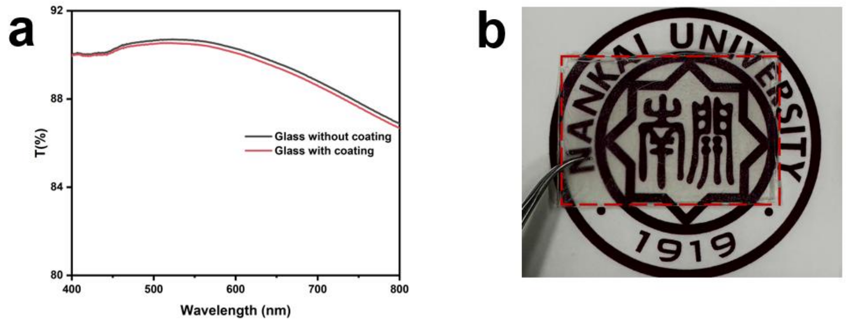

Supplementary Figure 23. (a) Transmittance test of glass coated with PU-UPy. (b) Photo image of PU-UPy film.

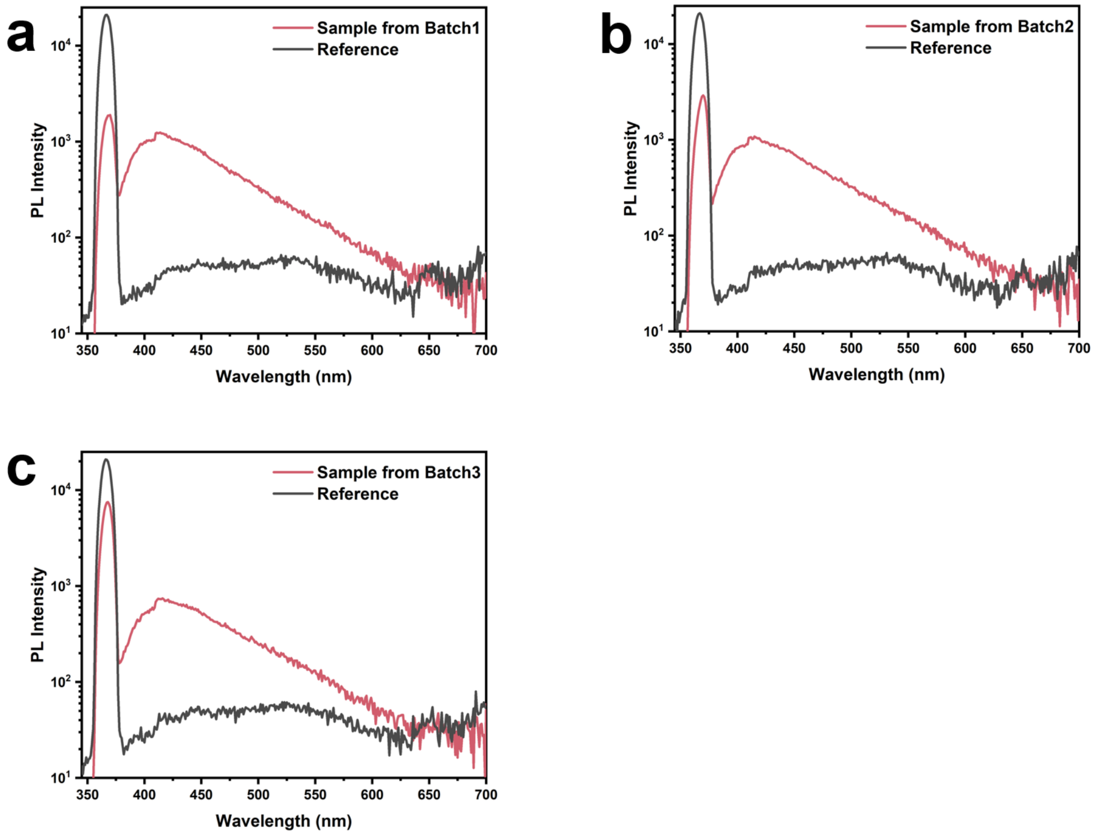

Supplementary Figure 24. PLQY testing of three different batches of PU-UPy (a-c).

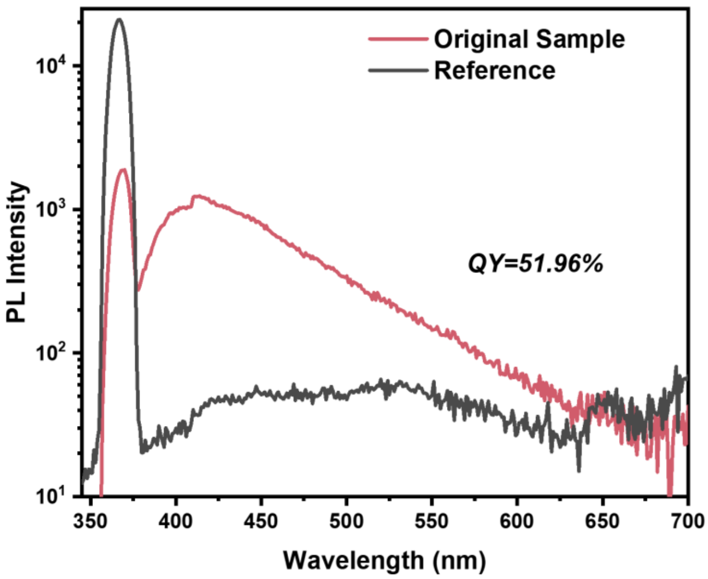

Supplementary Figure 25. PLQY test results of Original PU-UPy.

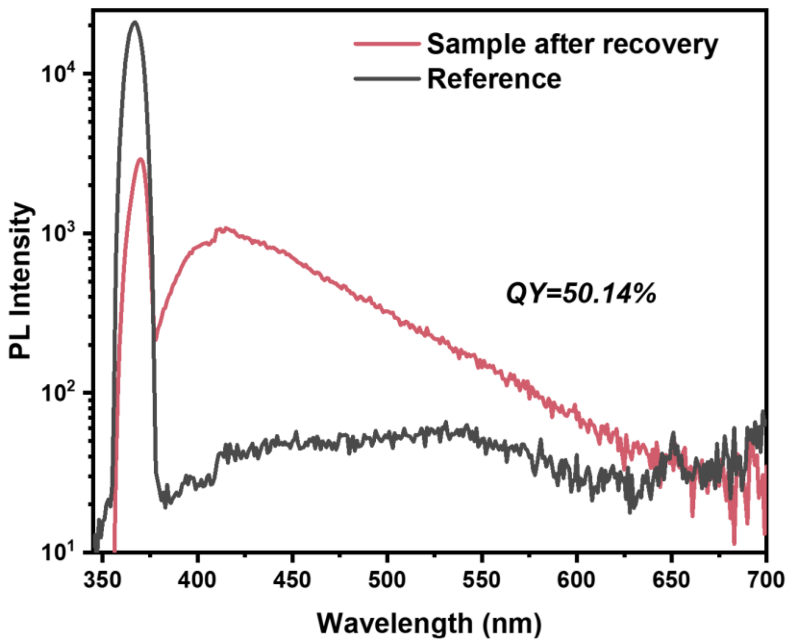

Supplementary Figure 26. PLQY test results of Reshaped PU after shredding.

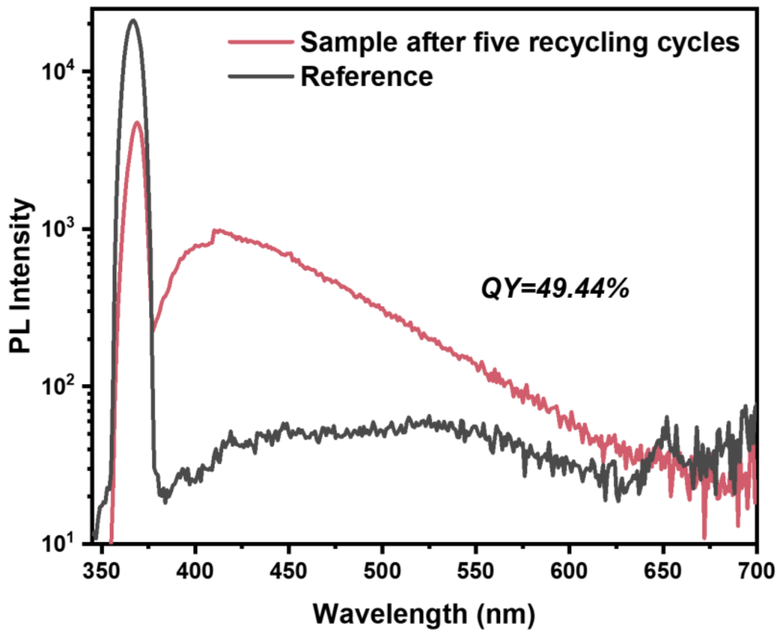

Supplementary Figure 27. PLQY test results of PU obtained after repeated shredding and remodeling five times.

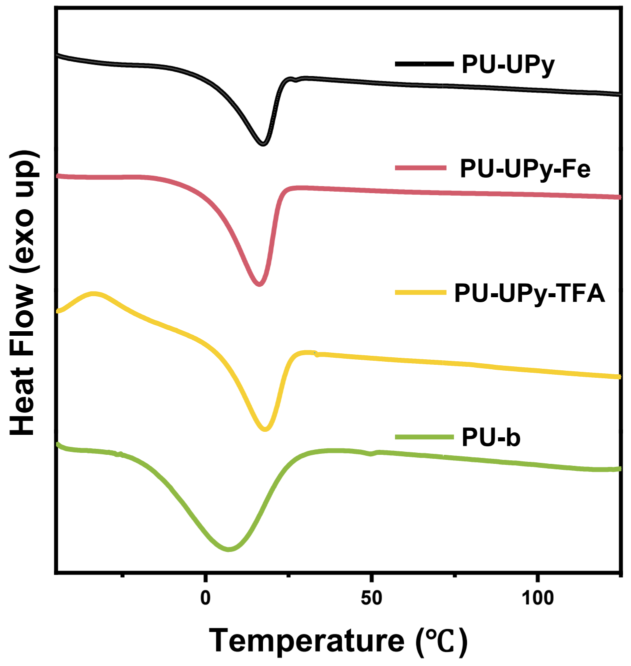

Supplementary Figure 28. DSC curves of PU-UPy, PU-UPy-TFA, PU-UPy-Fe and PU-b.

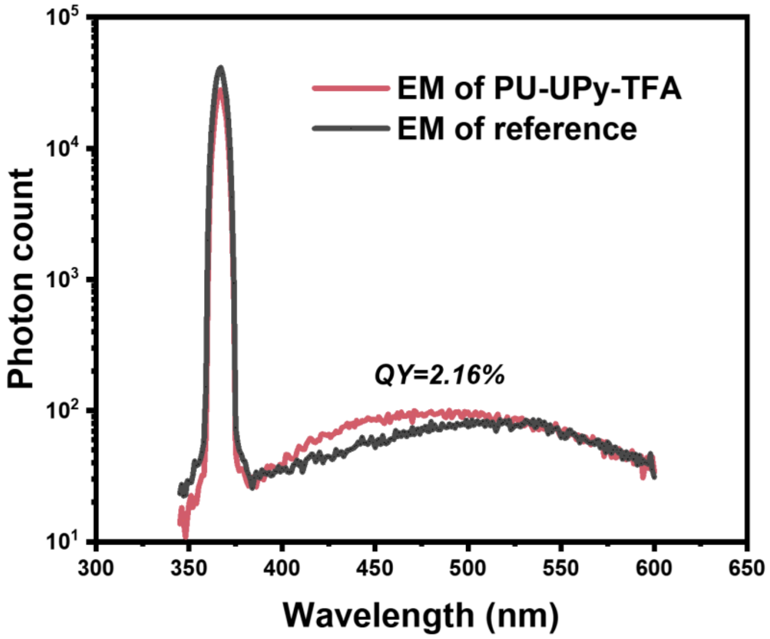

Supplementary Figure 29. PLQY test results of TFA-treated sample excited at the wavelength of 365nm.

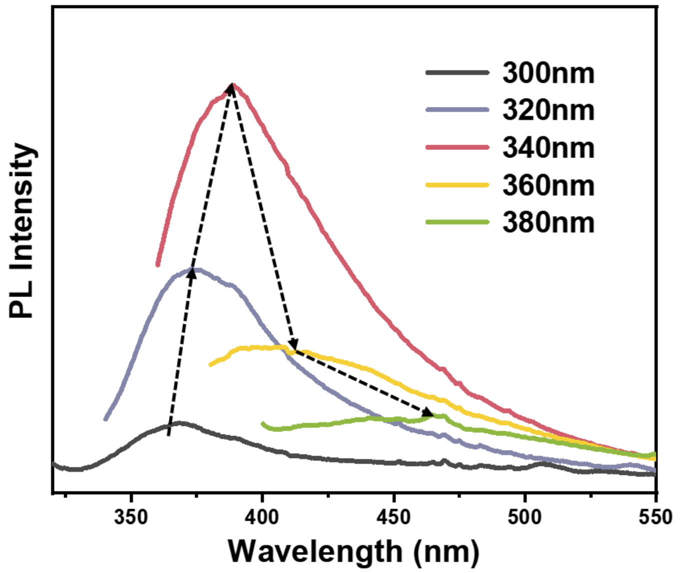

Supplementary Figure 30. Emission spectra of TFA-treated sample excited at different wavelength (The arrows in the figure indicate the change in the emission wavelength).

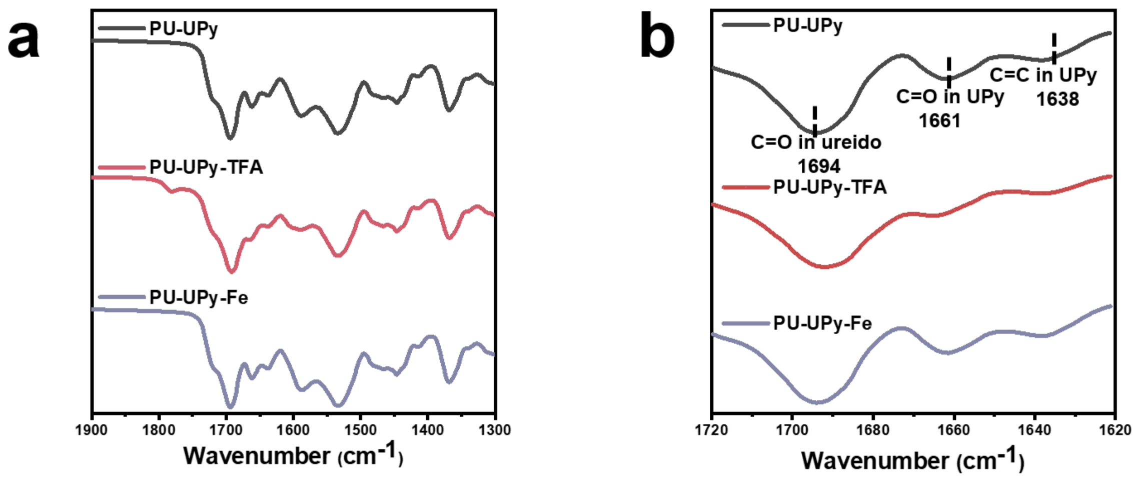

Supplementary Figure 31. (a) and (b) FTIR spectra of PU-UPy, PU-UPy-TFA and PU-UPy-Fe.

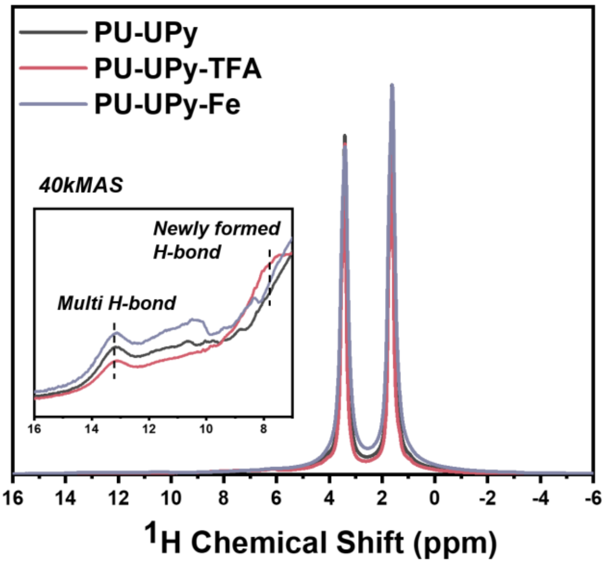

Supplementary Figure 32. ^{1}H-Solid state NMR spectra of the above samples.

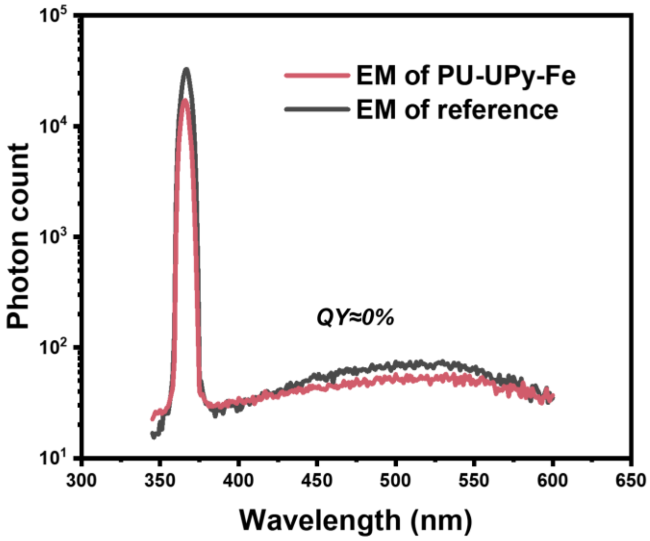

Supplementary Figure 33. PLQY test results of $FeCl_3$-treated sample excited at the wavelength of 365nm.

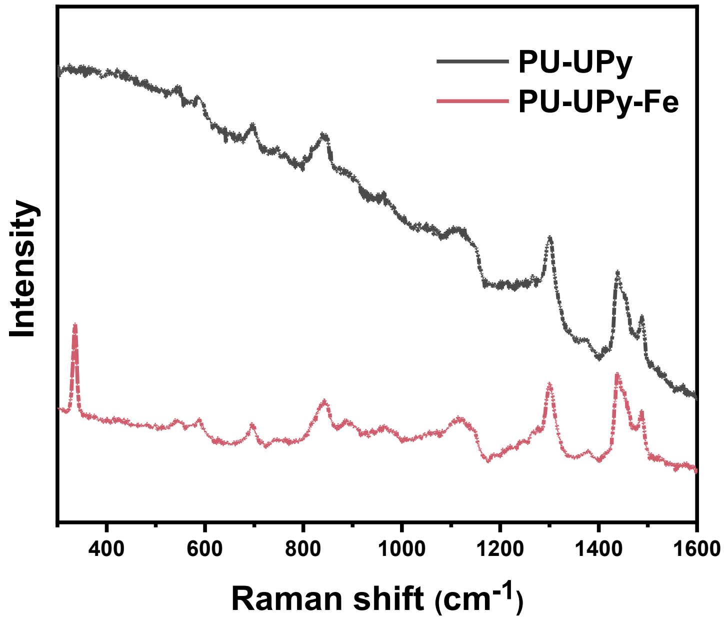

Supplementary Figure 34. Raman spectra of PU-UPy before and after treating with $FeCl_3$ aqueous solution.

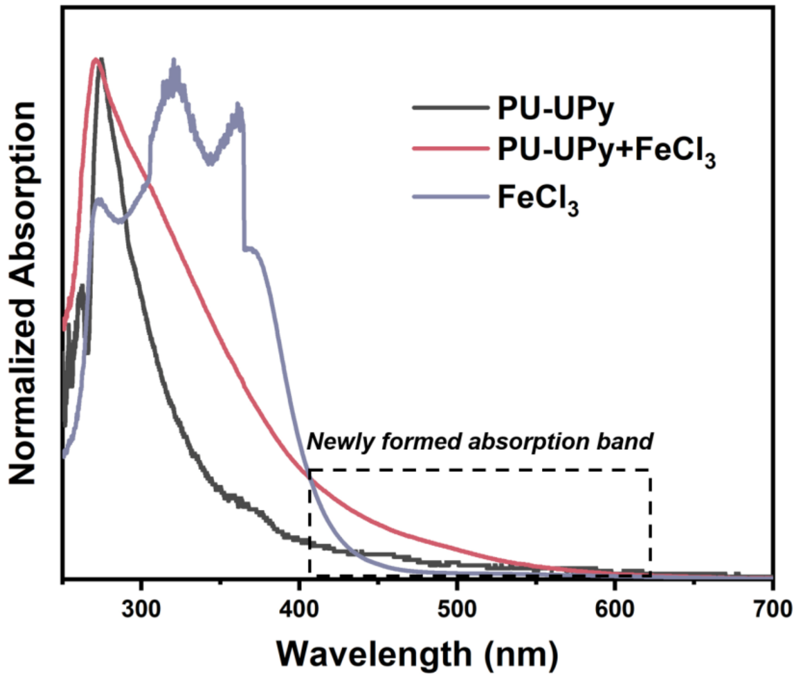

Supplementary Figure 35. Normalized UV-vis absorption spectra of PU-UPy, PU-UPy+$FeCl_3$ and $FeCl_3$ in DMF.

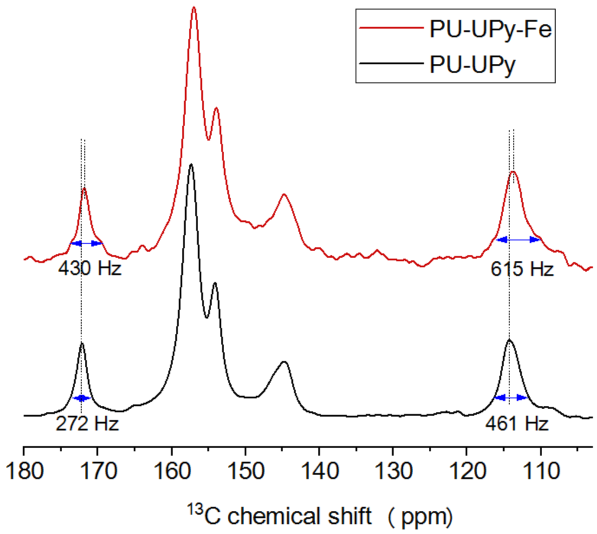

Supplementary Figure 36. Solid-state ^{13}C CPMAS NMR spectrum of PU-UPy and PU-UPy-Fe.

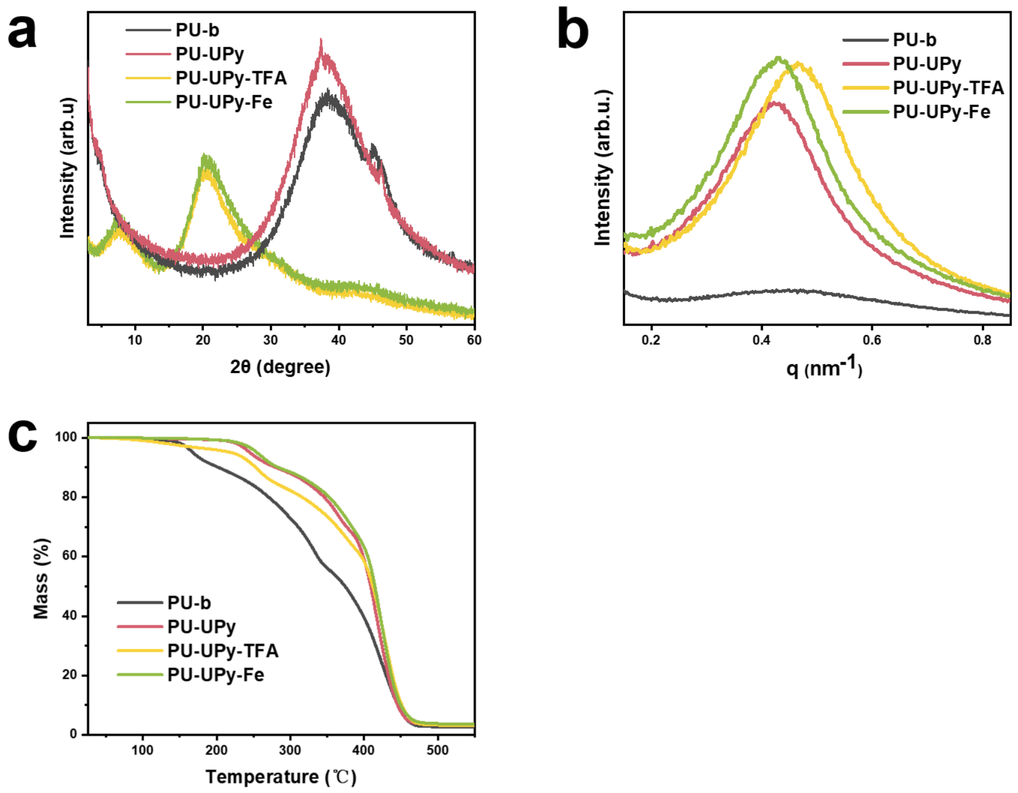

Supplementary Figure 37. XRD profiles (a), one-dimensional SAXS scattering spectrum and TGA curves of PU-UPy, PU-UPy-TFA, PU-UPy-Fe and PU-b samples.

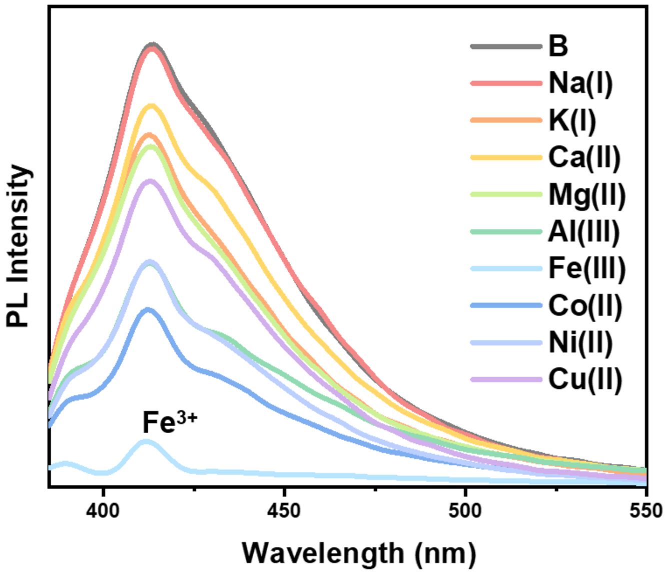

Supplementary Figure 38. PL spectra of PU-UPy (20 μM) in DMF with various metal ions (200 μM), emitted at the wavelength of 365 nm.

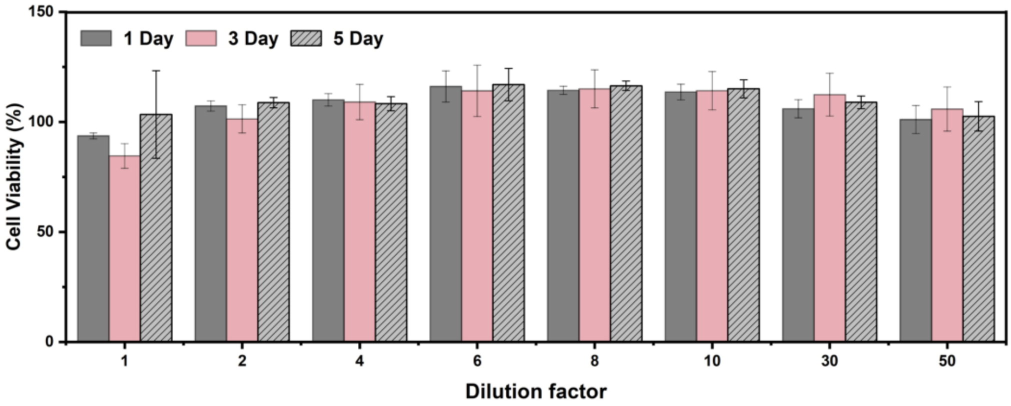

Supplementary Figure 39. Cell viability of different dilution factors of the experimental materials group (compared with the negative control group). The error bars in the figures represent the standard deviation derived from three independent replicate measurements for each data point. They were calculated as the positive square root of the sample variance for each set of triplicates and are displayed as ±1 SD.
